# Evaluation of a modified ultrasound-assisted technique for mid-thoracic epidural placement: a prospective observational study

**DOI:** 10.1186/s12871-024-02415-x

**Published:** 2024-01-19

**Authors:** Chanyan Huang, Ying Chen, Mengjia Kou, Xuan Wang, Wei Luo, Yuanjia Zhang, Yuting Guo, Xiongqing Huang, Lingzhong Meng, Ying Xiao

**Affiliations:** 1 Department of Anesthesiology, The First Affiliated Hospital, Sun Yat-sen University, No. 58, Zhongshan 2nd Road, Guangzhou, 510080 Guangdong China; 2 Department of Neurology, State Key Laboratory of Complex Severe and Rare Diseases, Peking Union Medical College Hospital, Chinese Academy of Medical Sciences and Peking Union Medical College, Beijing, China; 3 Department of Anesthesia, Indiana University School of Medicine, Indianapolis, IN USA

**Keywords:** Analgesia, epidural, Anesthesia, epidural, Anesthetics, local, Pain, postoperative, Ultrasonography, Thoracic surgery

## Abstract

**Background:**

Although mid-thoracic epidural analgesia benefits patients undergoing major surgery, technical difficulties often discourage its use. Improvements in technology are warranted to improve the success rate on first pass and patient comfort. The previously reported ultrasound-assisted technique using a generic needle insertion site failed to demonstrate superiority over conventional landmark techniques. A stratified needle insertion site based on sonoanatomic features may improve the technique.

**Methods:**

Patients who presented for elective abdominal or thoracic surgery requesting thoracic epidural analgesia for postoperative pain control were included in this observational study. A modified ultrasound-assisted technique using a stratified needle insertion site based on ultrasound images was adopted. The number of needle passes, needle skin punctures, procedure time, overall success rate, and incidence of procedure complications were recorded.

**Results:**

One hundred and twenty-eight subjects were included. The first-pass success and overall success rates were 75% (96/128) and 98% (126/128), respectively. In 95% (122/128) of patients, only one needle skin puncture was needed to access the epidural space. The median [IQR] time needed from needle insertion to access the epidural space was 59 [47–122] seconds. No complications were observed during the procedure.

**Conclusions:**

This modified ultrasound-assisted mid-thoracic epidural technique has the potential to improve success rates and reduce the needling time. The data shown in our study may be a feasible basis for a prospective study comparing our ultrasound-assisted epidural placements to conventional landmark-based techniques.

## Introduction

Thoracic epidural anesthesia is widely applied in thoracic and major abdominal surgical procedures because it provides excellent perioperative analgesia and reduces morbidity and mortality [1–7]. However, the placement of an epidural catheter, especially in the mid-thoracic region, is regarded as one of the most challenging procedures in anesthetic practice [8–10]. Technical difficulties often discourage its use. Reducing the technical difficulty of thoracic epidural placement is desirable, as multiple needle insertion attempts during a difficult thoracic epidural placement may increase the risk of complications and experiences of discomfort [11].

A previous study on preprocedural ultrasound examination to identify midline and interlaminar spaces for needle insertion showed no advantages in reducing the number of needle passes or needling time compared to the standard landmark technique [12]. One potential cause for this result may be that the generic textbook needle insertion point (1 cm caudal and 1 cm lateral to the intersection between the midline and interlaminar space skin mark) ignores the wide intersubject variability in anatomy [13]. Therefore, we speculate that stratification of the needle entry point according to the sonoanatomical features of the thoracic spine would facilitate epidural catheterization. Furthermore, the inaccuracy of skin markings, which is the main drawback of the preprocedural ultrasound technique, constrains the ability of ultrasound to demonstrate its value. The discrepancy between the skin marking and the deep structure appears if the initial needle insertion direction differs from the angle of the probe [14, 15]. Even minor unnoticeable caudad or cephalad angulation of the probe may significantly alter the precision of skin markings. However, there is limited research on optimizing patient position to improve the accuracy of skin markings.

In this article, we describe the practice of thoracic epidural placement under a modified ultrasound-assisted technique using a stratified needle insertion site and a modified position to improve the accuracy of skin markings. We showed the results of our technique in 128 consecutive patients regarding the feasibility of this technique.

## Materials and methods

This single-center, prospective observational study was conducted at a tertiary care academic medical center in Guangzhou, China. After receiving internal review board approval (Ref: [2022]139) and written informed consent, adult patients scheduled for thoracic or upper abdominal surgery and suitable for thoracic epidural analgesia between August 2022 and December 2022 were recruited. Exclusion criteria included contraindications to epidural catheterization, preexisting coagulopathy, localized infection, allergy to local anesthetics, patient refusal and pregnancy.

### Description of technique

**Fig. 1:**
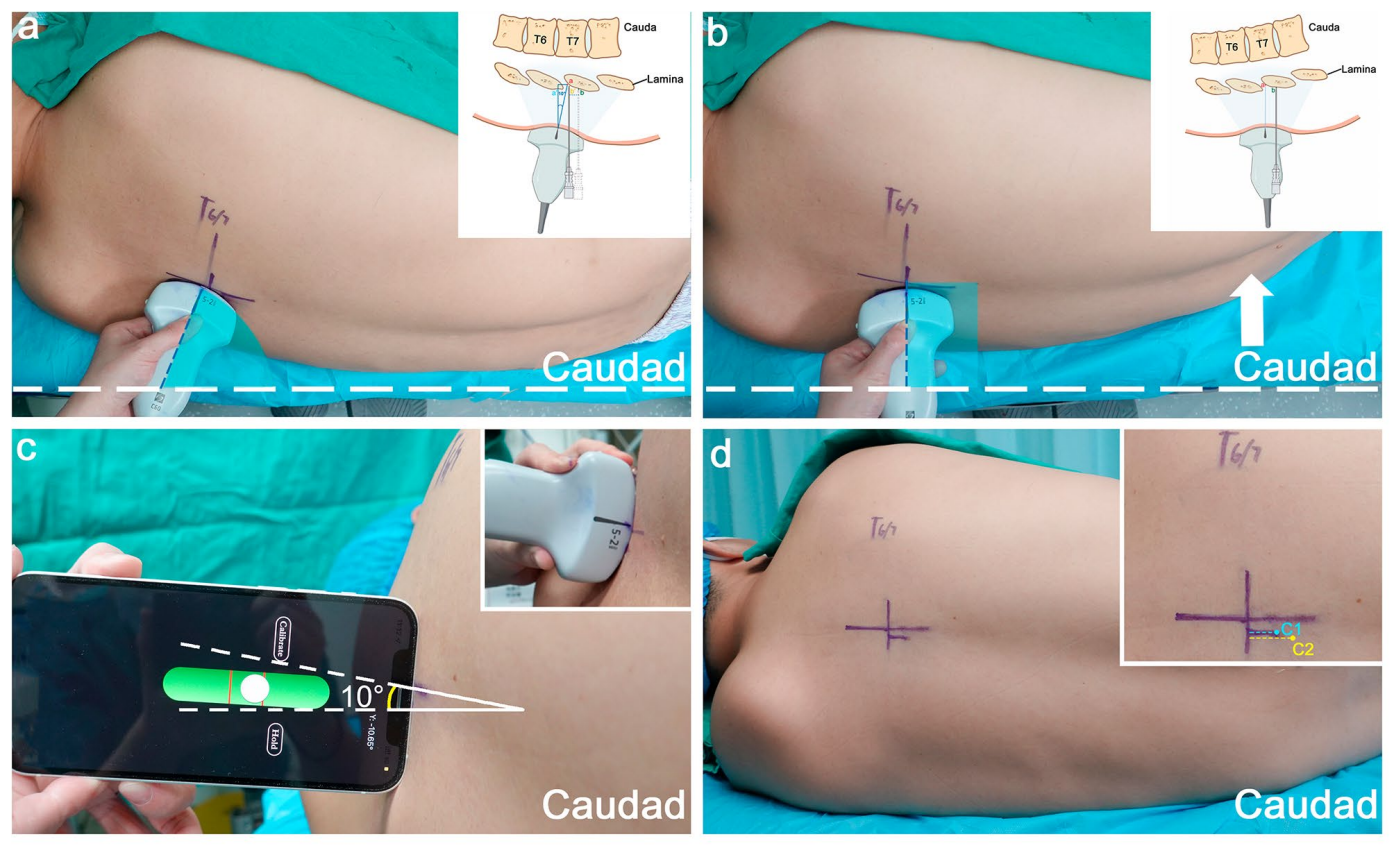
Steps of modified lateral decubitus position and skin markings for the needle entry point. (**a**) Standard left-lateral decubitus position with the shoulder and the hip being placed at the edge of the bed. To obtain an optimal paramedian sagittal oblique (PMSO) view with basically horizontal laminae, the probe was placed with a cephalad-to-caudal orientation, as indicated by the blue sector. The white dotted line represents the edge of the bed. (**b**) The hip was moved forward (illustrated by the white arrow) to achieve a perpendicularly (to the edge of the bed) placed probe with an optimal PMSO view, as indicated by the blue rectangle. The caudal edge of the T6/7 interlaminar space was marked on the overlying skin. (**c**) The back was pushed forward with a 10° anterior oblique, verified via an inclinometer tool on a smartphone. In the transverse median (TM) interlaminar view, the midline of spinous processes was marked on the skin. (**d**) Determination of the needle entry site, approximately 0.5–1 cm lateral to the midline and 1–1.5 cm caudal to the interlaminar space. The blue dot (C1) represents the needle entry point for patients stratified as Category I, 0.5 cm lateral to the midline and 1 cm caudal to the interlaminar space. The yellow dot (C2) represents the needle entry point for patients stratified as Category III, 1 cm lateral to the midline and 1.5 cm caudal to the interlaminar space

After establishing intravenous access, standard American Society of Anesthesiologists monitoring was applied throughout the thoracic epidural placement, including pulse oxygen saturation, electrocardiogram, and noninvasive blood pressure. All patients received supplemental oxygen via nasal cannula and intravenous sedation (dexmedetomidine 20 µg and sufentanil 5 µg). Patients were positioned in the left lateral decubitus position with their hips and knees flexed as much as possible. The shoulder and pelvis were positioned at the edge of the bed, parallel to one another and perpendicular to the surface of the bed (Fig. 1a).

The targeted epidural level was determined based on the scheduled surgery. Preprocedural ultrasound scanning was performed using a 5-2-MHz frequency curved probe (Fujifilm Sonosite, Inc., Bothell, WA, USA). The probe was placed in the parasagittal plane approximately 5 cm from the midline. The interspace was identified using either the counting-up method from the twelfth rib or the counting-down method from the first rib until the targeted level was identified in the paramedian sagittal oblique (PMSO) view (Fig. 1a). The probe was then moved medially to identify the lamina and the anterior complex (AC). According to the initial craniocaudal angulation of the probe required to obtain a basically horizontal laminae image, we adjusted the patient’s position to ensure that the probe was placed perpendicularly to the edge of the bed, with the pelvis pushed forward to eliminate caudal angulation of the probe or the shoulder moved forward to eliminate cranial angulation (Fig. 1b). In this PMSO view, the caudal border of the interlaminar space was marked on the overlying skin, and the skin-to-lamina depth was measured when the probe was held against the skin with minimal compression. Then, we pushed the back forward with a small degree anterior oblique of 5–10°, verified via a clinometer (Fig. 1c).

**Fig. 2:**
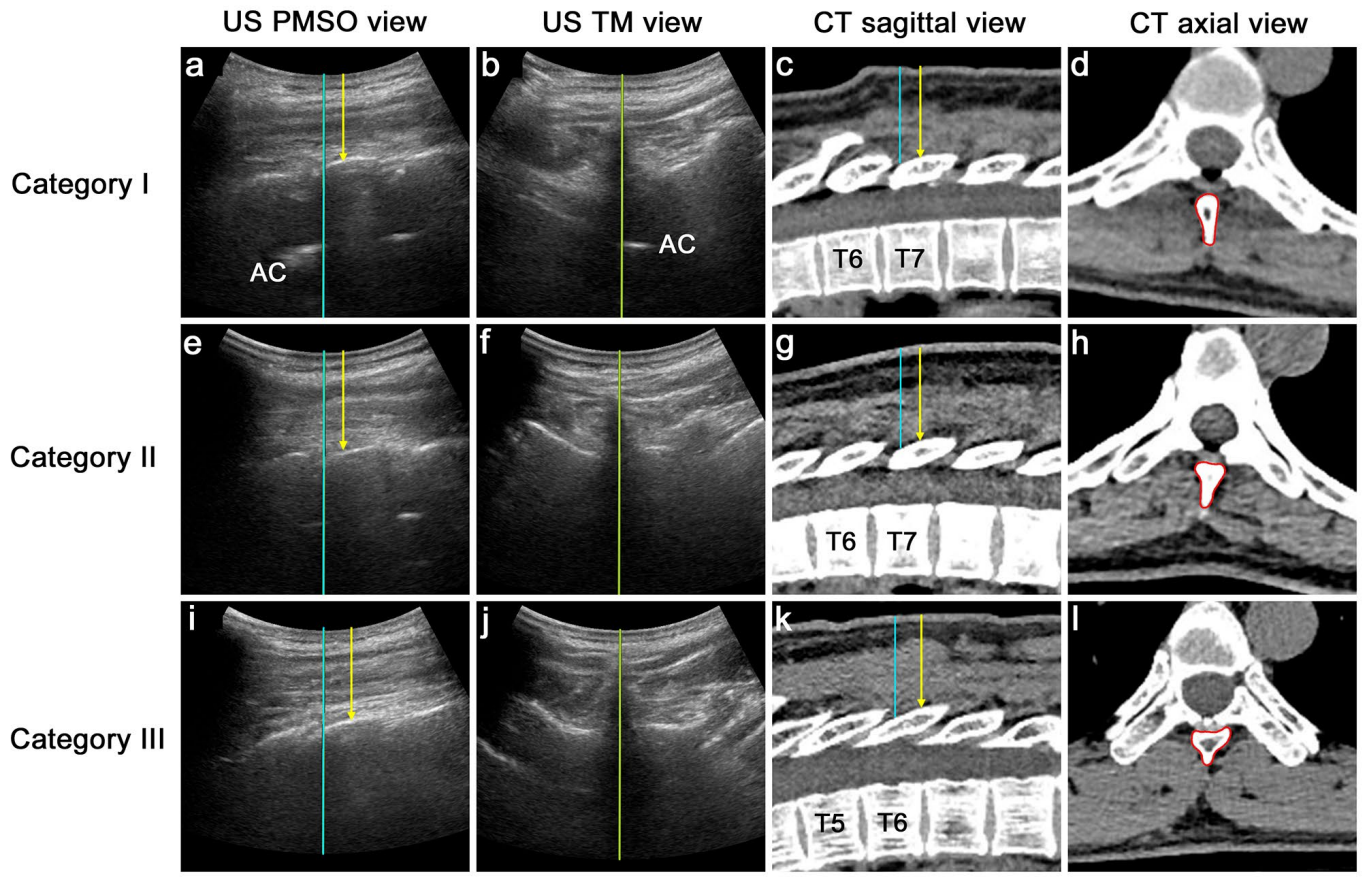
Paramedian sagittal oblique and transverse median views of the mid-thoracic spine and corresponding computed tomography image illustrating patient stratification. Category I: AC is visible in the PMSO and TM views (**a, b**). Note that the adjacent laminae are not closely overlapping (**c**), and the interlaminar space is not obstructed by the skinny-based spinous process (**d**). Category II: AC is only visible in the PMSO view (**e**) but invisible in the TM view (**d**). Note that the adjacent laminae are not closely overlapping (**g**), and the medial part of the interlaminar space is covered by the wide-based spinous process, leaving only the lateral portion of the interlaminar space open (**h**). Category III: AC is invisible in both views (**i, j**). Note that the closely overlapping laminae narrowed the interlaminar space (**k**), and the wide-based spinous process obstructed the interlaminar space (**l**). The blue lines represent the caudal edge of the interlaminar space of the chosen level. The yellow arrows represent the skin-to-lamina depth at the determined needle puncture site. The yellow lines represent the midline of the spinous process. The red line encircled areas represent the base of the spinous process. (AC = anterior complex; CT = computed tomography; PMSO = paramedian sagittal oblique; TM = transverse median)

The probe was then moved 90° to obtain the transverse median (TM) interlaminar view and inclined slightly in the cephalad direction to obtain the view of the vertebral canal of the chosen interlaminar space, although the AC in the mid-thoracic spine was not always visible. The midline of the spinous process was centered on the screen and marked on the skin. A stratified needle insertion site was adopted, 0.5 cm or 1 cm lateral to the midline and 1 cm or 1.5 cm caudal to interlaminar space (Fig. 1d), determined by the visibility of AC in the PMSO and TM views and the depth of laminae. (Table 1; Fig. 2). Patients were classified into three categories based on ultrasound images: (1) category I: AC visible in both the PMSO and TM views; (2) category II: AC visible only in the PMSO view but not the TM view; and (3) category III: AC invisible in either the PMSO view or TM view.

**Table 1:** Needle entry point determination based on patient stratification using anatomical features offered by ultrasound and illustrated by CT scans

Category	AC visible		Explanation via CT	Needle entry point	
	PMSO	TM		Lateral offset	Caudal shift
I	Yes	Yes	Skinny-base SP with wide IS; partially overlapping adjacent laminae	0.5 cm	1 cm
II	Yes	No	Slanted wide-base SP covering part of IS; partially overlapping adjacent laminae; lateral part of IS open	1 cm	1 cm: depth of lamina<4 cm; 1.5 cm: depth of lamina ≥ 4 cm
III	No	No	Steeply slanted wide-base SP obstructing IS; IS obstructed by closely overlapping lamina	1 cm	1.5 cm

Abbreviations: AC = anterior complex; CT = computed tomography; IS = interlaminar space; PMSO = paramedian sagittal oblique; SP = spinous process; TM = transverse median

All epidural procedures were performed with a 17-gauge Tuohy needle and a 19-G flex-tip catheter (Flex-Tip Plus, Arrow International, Reading, Pennsylvania) by one of the two attending anesthesiologists (YX or CH), who were experienced in ultrasound imaging of the spine. The Tuohy needle was initially inserted perpendicularly to the edge of the bed to contact the lamina and then walked off the lamina cranially without medial angulation (if AC was visible in TM view) or slight medial angulation (if AC was not visible in TM view) until the needle tip was engaged in the ligamentum flavum. During the epidural placement procedure, the bony contact with the lamina of the vertebra below acted as a depth finder. If bony contact occurred at a more superficial depth than that measured by ultrasound, the landing spot might be more medial than expected; no medial angulation was needed when walking off the lamina. In contrast, if the depth of bone contact is greater than the measured depth, the landing spot could be more lateral than expected; therefore, medial angulation was adopted when approaching the epidural space.

When the needle tip was situated in the ligamentum flavum, the loss of resistance (LOR) syringe was attached to the Tuohy needle hub. Then, the needle-syringe assembly was slowly advanced, and the LOR to air was intermittently elicited to confirm the entry of the epidural space. If it was impossible to access the epidural space due to multiple bony obstructions (> 3 minimal needle manipulations with different directions), then the needle-syringe assembly was partially withdrawn and readvanced with a different obliquity. Up to three needle redirections at each needle pass and a maximum of three passes were allowed in each skin puncture. If epidural access failed after three passes, a second skin puncture site, 0.3 cm caudal to the first skin puncture site, was adopted as suggested by a previous fluoroscopic-guided technique [16, 17]. If the epidural space could not be accessed despite three passes at the second needle insertion point, an alternative interlaminar space was used.

**Fig. 3:**
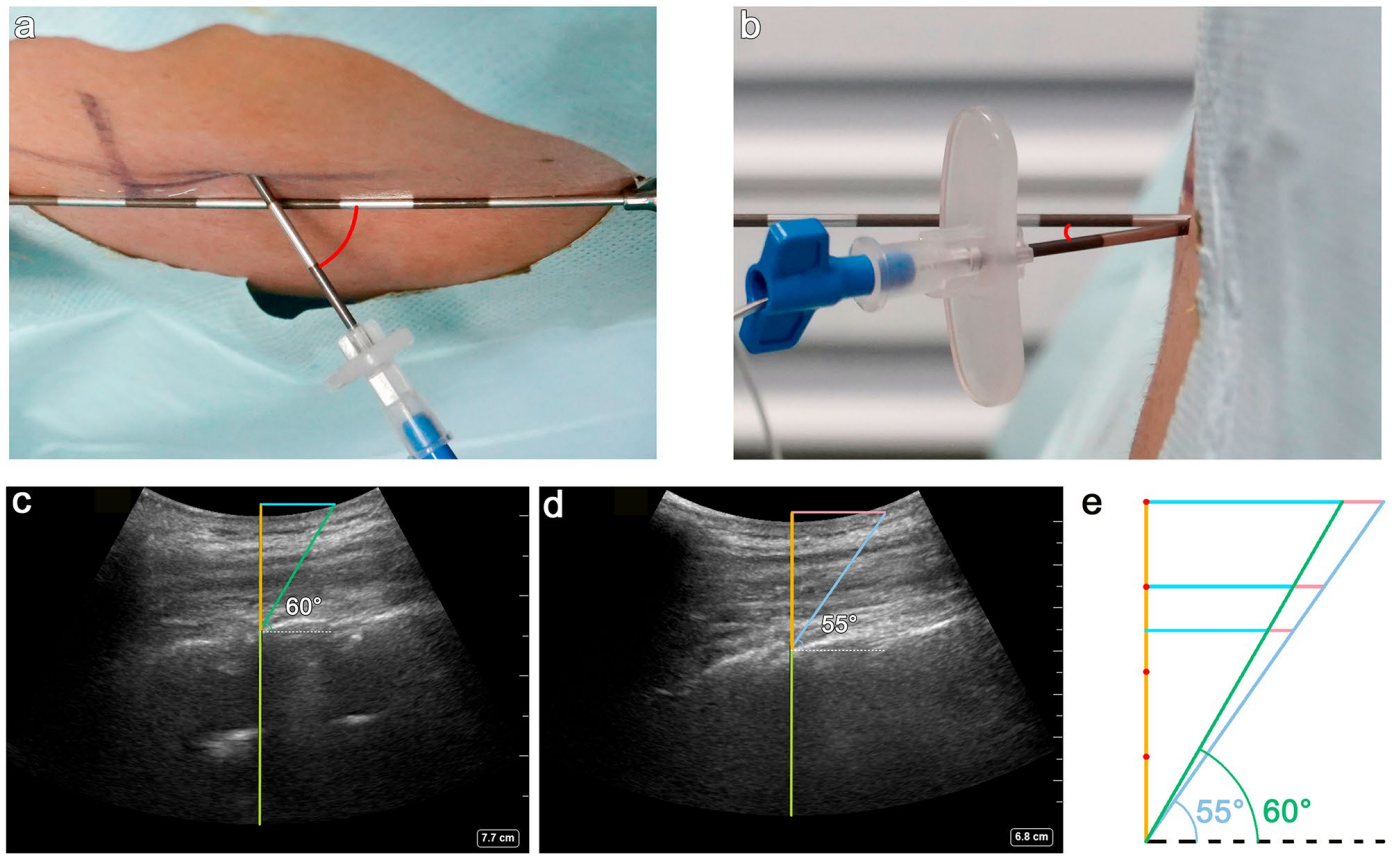
Needle insertion angle measurements and ultrasound images illustrating the optimal needle insertion angle. (**a**) Photo (from superior-to-inferior) showing the measurement of the upward angulation, which represents the craniocaudal angulation of the needle in the sagittal plane. An epidural needle was held along the long axis of the spine, representing the line made by the posterior border of the vertebral body. (**b**) The photo (from left to right) showing the measurement of the inward angulation, which represents the lateral to medial angulation of the needle in the transverse plane. An epidural needle was held horizontally, representing the horizontal plane of the line of the spinous process. (**c**) The paramedian sagittal oblique view illustrating a less acute angle (60°) of needle trajectory is optimal for patients with wide interlaminar space (anterior complex visible). (**d**) The paramedian sagittal oblique view illustrating a more acute (55°) needle trajectory is adopted for patients with closely overlapping laminae (anterior complex not visible). (**e**) The trigonometry formula illustrating that, as the needle trajectory becomes more acute or the skin-to-lamina depth increases, the caudal shift should be increased

Once LOR was obtained, the catheter was advanced smoothly without any resistance, and 4–5 cm remained in the epidural space. The epidural needle insertion angles in both the sagittal (upward angulation) and transverse (inward angulation) directions were measured based on two photos of the inserted needle, one from superior-to-inferior and one from left-to-right (Fig. 3a-b). A video camera was used to record the epidural placement process, and a blinded investigator reviewed the video at a later time.

### Outcome measures

Patient demographics and clinical data were obtained from the electronic medical records. The estimated depth of the lamina was measured via a built-in caliper in the ultrasound machine. The actual needle depths extending from the skin entry point to the lamina and the epidural space were recorded.

Outcome measures included: (1) first-pass success, defined as achievement of LOR and successful catheter placement through a single skin puncture with no needle withdrawal and redirection; (2) number of needle passes: needle tip maneuvers toward the midline and cephalad were considered standard needle walking techniques and counted as a single pass; an additional needle pass was defined as the needle returning to the initial insertion direction (perpendicular to the edge of the bed) before reinsertion; (3) first-attempt success, defined as the needle achieving LOR and successful catheter placement through a single skin puncture; (4) number of attempts, defined as the number of complete needle withdraw and reinsertion through a new location at the same or an alternate level; (5) overall success rate; (6) needling time, defined as the initial insertion of the Tuohy needle into the skin until the final LOR; (7) patient satisfaction, measured after the epidural placement using a five-point Likert-type scale: 1 = very satisfied, 2 = satisfied, 3 = average, 4 = unsatisfied, 5 = very unsatisfied.

We evaluated clinical success through dermatomal coverage and analgesic effects and recorded procedure-related complications, including epidural hematoma, vasovagal reaction, inadvertent dural puncture, pneumothorax, intravascular or intrathecal local anesthetic injection, and spinal cord injury.

### Statistical analysis

The normality of the data distribution was assessed using the Shapiro‒Wilk test. Continuous data were reported as the mean ± standard deviation (SD) with range or median (interquartile range [IQR]) with range and were analyzed with the two-sample t test or Mann‒Whitney U test, respectively. Categorical variables were reported as numbers (percentages) and analyzed with the Pearson chi-square test or Fisher’s exact test where appropriate. All statistical analyses were performed using JMP Pro 16.2.0 software (SAS Institute Inc., Cary, NC, USA). A two-sided *p* value of less than 0.05 was considered statistically significant.

## Results

During the study period, a total of 128 patients were included for analysis, with a mean ± SD age of 59 ± 18 years. The T5-6 and T6-7 levels were chosen for nearly half of the needle insertions (59/128). (Table 2)

**Table 2:** Demographics and clinical characteristics of patients

Variable	Total (*n* = 128)
Age, years	59.3 ± 11.7, (range: 29–86)
Sex	
Male	67 (52%)
Female	61 (48%)
Weight, kg	59.6 ± 9.8, (range: 39–87)
Height, m	1.6 ± 0.1, (range: 1.4–1.9)
BMI, kg/m^2^	22.5 ± 3.1, (range: 15.8–31.6)
Type of surgery	
Lung	58 (45%)
Hepatobiliary	22 (17%)
Gastrointestinal	18 (14%)
Esophagectomy	16 (13%)
Pancreatic	14 (11%)
Level of puncture	
T5-6	18 (14%)
T6-7	41 (32%)
T7-8	26 (20%)
T8-9	43 (34%)
Category based on ultrasound imaging ^a^	
Category I	48 (38%)
Category II	50 (39%)
Category III	30 (23%)

Data are presented as numbers (percentages) or means ± SDs with ranges

Abbreviations: BMI = body mass index; IQR = interquartile range; SD = standard deviation

^a^ Category I, II and III indicate that the anterior complex was visible in both the paramedian sagittal oblique view and transverse median view, visible only in the paramedian sagittal oblique view, and invisible in both views, respectively

Descriptive statistics of procedure variables and outcomes are summarized in Table 3. The median [IQR] needle insertion depth was 5.0 [4.5–5.5] cm, with a mean ± SD upward angulation of 61° ± 8° and a median [IQR] inward angulation of 10° [8–13°]. First-pass success was achieved in 75% (96/128) of patients. In 95% (122/128) of patients, only one needle skin puncture was needed to access the epidural space. The overall success rate was 98% (126/128). The median [IQR] time needed from needle insertion to access the epidural space was 59 seconds [47–122 seconds]. A total of 118 patients (92%) found the epidural insertion performance to be satisfactory or very satisfactory. No procedure-related complications were observed. All patients reported appropriate dermatomal coverage and adequate analgesia.

**Table 3:** Characteristics and outcomes of mid-thoracic epidural placement

Variable	Total (*n* = 128)
**Characteristics**	
Estimated skin-to-lamina depth ^a^, cm	3.0 [2.5–3.2], (range: 2.0–4.5)
Actual skin-to-lamina depth, cm	3.0 [2.5–3.5], (range: 2.0–4.5)
Skin-to-LOR depth, cm	5.0 [4.5–5.5], (range: 3.5–6.5)
Needle insertion angles, °	
Upward angulation ^b, c^	61 ± 8, (range: 38–78)
Inward angulation ^b, d^	10 [8–13], (range: 0–18)
**Outcomes**	
First-pass success rate ^e^	96 (75%)
First-attempt success rate ^f^	122 (95%)
Overall success rate	126 (98%)
Needling time, seconds ^b, g^	59 [47–122], (range: 28–533)
Number of needle passes ^b^	1 [1–1], (range: 1–6)
Number of attempts ^b^	1 [1–1], (range: 1–2)
Patient satisfaction	
1 (very unsatisfied)	0
2	4 (3%)
3 (neutral)	6 (5%)
4	26 (20%)
5 (very satisfied)	92 (72%)

Data are presented as numbers (percentages), means ± SDs with ranges, or medians [IQRs] with ranges

Abbreviations: IQR = interquartile range; LOR = loss of resistance; SD = standard deviation

^a^ The estimated skin-to-lamina depth was measured via a built-in caliper in the ultrasound machine

^b^ Data were not available for two patients with epidural puncture failures

^c^ Upward angulation represents the craniocaudal angulation of the needle in the sagittal plane

^d^ Inward angulation represents lateral to medial angulation of the needle in the transverse plane

^e^ First-pass success, defined as achievement of LOR and successful catheter placement through a single skin puncture with no needle withdrawal and redirection

^f^ First-attempt success, defined as the needle achieving LOR and successful catheter placement through a single skin puncture

^g^ Needling time, defined as the time from epidural needle insertion to successful access of the thoracic epidural space via LOR.

As presented in Table 4, compared to patients with AC visible in the PMSO view (category I and II), those with AC invisible (category III) had a higher first-pass success rate (86% vs. 40%, *P* < 0.001), higher first-attempt success rate (99% vs. 83%, *P* = 0.003), and significantly shorter median needling time (57 seconds vs. 156 seconds, *P* < 0.001).

**Table 4:** Comparison of different categories based on sonoanatomy on outcomes

Variable	Category I and II ^a^ (*n* = 98)	Category III ^b^ (*n* = 30)	*P* value
First-pass success rate ^c^	84 (86%)	12 (40%)	<0.001
First-attempt success rate ^d^	97 (99%)	25 (83%)	0.003
Overall success rate	98 (100%)	28 (93%)	0.054
Needling time, seconds ^e^	57 [44–77]	156 [61–245]	<0.001
Number of needle passes	1 [1–1]	2 [1–3]	<0.001
Number of attempts	1 [1–1]	1 [1–1]	0.011

Data are presented as numbers (percentages) or medians [IQRs].

^a^ Category I-II indicates that the anterior complex was visible in the paramedian sagittal oblique view

^b^ Category III indicates that the anterior complex was invisible in both the paramedian sagittal oblique view and transverse median view

^c^ First-pass success, defined as achievement of LOR and successful catheter placement through a single skin puncture with no needle withdrawal and redirection

^d^ First-attempt success, defined as the needle achieving LOR and successful catheter placement through a single skin puncture

^e^ Needling time, defined as the time from epidural needle insertion to successful access of the thoracic epidural space via LOR.

## Discussion

We described the efficacy of a modified ultrasound-assisted paramedian approach for thoracic epidural placement. The use of a stratified needle insertion point according to sonoanatomical categorization, improving the precision of skin markings and minimizing medial angulation of needle trajectory are crucial technical components to improve the success rate.

Difficulty in performing thoracic epidural placement may decrease procedure efficiency, and it may result in anesthesiologists abandoning the procedure despite the possible advantage of epidural analgesia for the patients. Technological improvements are desirable for thoracic epidural placement. Currently, the conventional landmark technique is the most widely used for epidural placement. Compared with previous studies using the conventional surface landmark technique, we achieved a much higher first-pass success rate (75% vs. 35% [18]) and first-attempt success rate (95% vs. 60% [12]). Preprocedural ultrasound scans for epidural placement have been reported in recent studies, but the results did not always demonstrate superiority over conventional landmark techniques [12, 19]. However, using our modified ultrasound-assisted technique, first-pass success was achieved in 75% of patients, which was higher than that reported recently by Arzola et al. (39%) [19]. Potential explanations for the high success rates with the first pass and/or first attempt are as follows.

First, unlike any of the previously reported ultrasound-assisted techniques, we adopted a stratified needle insertion point based on the sonoanatomic categorization of the ultrasound image and the skin-to-lamina depth. Superior visibility of the AC in both the PMSO and TM views indicates a wider interlaminar space necessitating a less acute (60° as recommended [20]) passage of the epidural needle (Fig. 3c). The nonvisibility of the AC due to the extremely caudad angulation of wide-based spinous processes and closely overlapping laminae requires a more acute (e.g., 55°) passage and thus a more caudad needle entry point, as indicated by trigonometry (Fig. 3d). Thus, different from the conventional recommended fixed needle insertion site (1 cm caudal shift to interlaminar space), we adopted a more caudad insertion site (1.5 cm) for those with invisible AC and/or a depth of lamina > 4 cm. As the depth of the lamina increases, so should the caudal shift if the needle is advanced at a particular angle (Fig. 3e). Our results showed that stratified needle insertion points based on anatomical features could improve procedure efficiency.

**Fig. 4:**
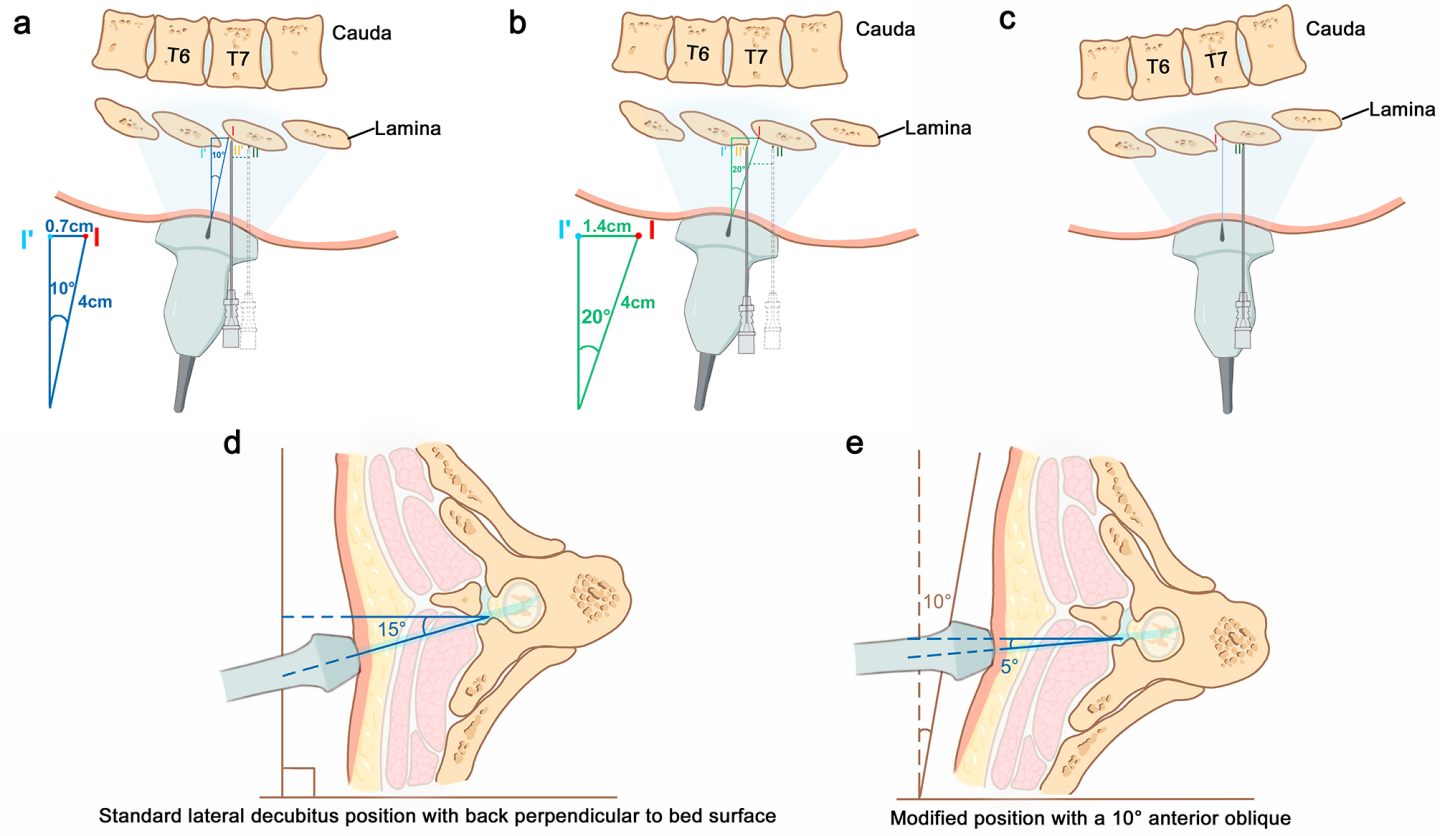
Schematic images illustrating a modified lateral decubitus position and the precise skin markings. (**a-b**) Geometrical representation of probe angulation and resultant inaccuracy of markings. If the ultrasound probe is angled 10° caudad and the skin-to-laminar depth is 4 cm, the location corresponding to the skin marking (Point I’) in a perpendicular direction was 0.7 cm (4 × Sin10 = 0.7) cranial to the interlaminar space (Point I). Consequently, the actual landing spot (Point II with the solid needle) was cranial to the intended spot (Point II’ with the dotted needle) and too close to the interlaminar space. If the probe had a 20° caudad angulation and the skin-to-laminar depth is 4 cm, the corresponding skin marking (Point I’) was 1.4 cm (4 × Sin20 = 1.4) cranial to the interlaminar space (Point I), rendering the landing spot crossover the target interlaminar space. (**c**) Schematic images illustrating that skin marking only works well when the probe angulation is the same as the initial angulation of needle advancement. (**d-e**) Schematic image showing the relationship between needle trajectory and positioning. The conventional paramedian approach under the standard lateral decubitus position is to introduce the needle at a lateral-to-medial angulation of approximately 15°. The medial angulation can be reduced with a 10° anterior oblique position

Second, unawareness about the sources of the inaccuracies of skin markings may also contribute to the remaining low first-pass success rate even with the aid of ultrasound in previous studies. Skin markings only work well when the initial needle angulation is the same as the ultrasound beam angulation. Because the thoracic spine is often a marked curved plane rather than a flat plane, especially when the patient is asked to flex as much as possible to widen the interlaminar space, it is easy to inadvertently rock the probe to obtain an optimal image of the PMSO view, which leads to craniocaudal angulation of the ultrasound beam. These small angulations, which might occur unnoticeably, would result in relatively large marking deviations when the angulations are not replicated by needle insertion. This implies that, for example, angulating a probe by 10° or 20° caudad would displace the marking approximately 7 mm or 14 mm cephalad at a depth of 4 cm when the needle was advanced perpendicular to the edge of the bed (Fig. 4a-b). We chose the line (the edge of the bed) as the reference because the patient’s back could be a curved plane. Marking deviation increases as the angulation of the probe increases. As the lamina depth increases, so does the error. Consequently, ignoring the probe’s angulation may result in an inappropriate landing spot, either too close to or crossing over the intended interlaminar space, making the landing spot unpredictable (Fig. 4a-b). The allowable degree of error varies with the size of the interlaminar space. This is why thoracic epidural placement for patients with nonvisible AC (category III) was more difficult than for those with visible AC (categories I and II), as indicated by the lower first-pass success rate (40% vs. 86%), lower first-attempt success rate (83% vs. 99%), and longer median needling time (156 seconds vs. 57 seconds). In our study, we eliminated caudal or cranial probe angulation by modifying the patient position so that the probe could be placed perpendicularly to the bed edge, the same as the initial direction of epidural needle advancement (Fig. 4c). This modification improved the accuracy of skin markings to represent the deep structures, which ultimately helped to demonstrate the benefit of ultrasound.

Additionally, we adopted a modified lateral decubitus position by 5–10° anterior oblique to minimize the medial angulation of the needle path (Fig. 4d-e). In our study, the median inward angulation was 10°, which was smaller than that reported using the fluoroscopic-guided technique (24.8° [21] and 35° [22]). Our minor inward angulation may contribute to the slightly higher first-pass success rate (75% vs. 34–68%) and shorter needling time (59 seconds vs. 95–123 seconds) than that of the fluoroscopic-guided epidural access reported in recent work [16]. An entry point that is too lateral invariably necessitates more medial angulation, which might increase the needle redirection process and thus decrease the first-pass success rate. Minimizing the inward angulation of the needle could facilitate a straight and predictable course in the epidural space with minimal risk of coiling during catheter insertion, reduce the risk of misadventure of the needle tip to the contralateral side, and allow traversing a minimal amount of erector spinal muscles during needle insertion to add to the comfort experience of the patient [23–26].

There are some limitations to our study. The observational nature of the study prevented us from making robust comparisons between patients receiving our modified ultrasound-assisted epidural placement and those receiving conventional landmark palpation techniques. A prospective, randomized study comparing these two techniques would be needed to confirm the benefits of our technique. However, evidence has shown that a well-designed observational study produces results that are analogous to randomized control trials or meta-analyses [27, 28]. In addition, the time needed for epidural catheterization depends on the performers’ experience. The performance of all the procedures by two experienced anesthesiologists may have led to all epidurals being placed more efficiently in this study; however, it decreases the generalizability. Future studies could address these issues by including other anesthesiologists with different expertise levels. Finally, the conventional endpoint (LOR to air) lacks specificity [29, 30]. Correct identification of the thoracic epidural space should be confirmed by objective modalities such as neurostimulation and waveform analysis [31–33]. We did not use any alternative technique to confirm the correct epidural catheter placement. However, we evaluated clinical success through dermatomal coverage and adequate analgesia with minimum opioid requirement in all patients.

In conclusion, this study demonstrated that the modified ultrasound-assisted paramedian approach for thoracic epidural placement is a feasible and promising technique. Given its high success rates within a superior procedural time frame, this approach could be utilized routinely to simplify thoracic epidural placement in clinical practice. Further studies are warranted to compare the performance of our modified ultrasound-assisted epidural placement to conventional landmark-based techniques.

## Data Availability

The datasets used and/or analyzed during the current study are available from the corresponding author upon reasonable request.

## Abbreviations

AC: anterior complex
CT: computed tomography
IQR: interquartile range
LOR: loss of resistance
PMSO: paramedian sagittal oblique
SD: standard deviation
TM: transverse median
